# Whole-body biomechanical and lifestyle predictors of pediatric musculoskeletal pain: a multi-center cross-sectional study

**DOI:** 10.3389/fpubh.2026.1689311

**Published:** 2026-03-09

**Authors:** Saleh M. Kardm, Abdulmohsen Saeed Kardm, Ziad Ahmed Alanazi, Tariq Abdullah Aldugman, Ravi Shankar Reddy, Ajay Prashad Gautam, Turki Ahmed Alqahtani

**Affiliations:** 1 Department of Surgery, College of Medicine, Najran University, Najran, Saudi Arabia; 2 Aseer Central Hospital, Abha, Saudi Arabia; 3 Orthopedic Division, Department of Surgery, College of Medicine, Jouf University, Sakaka, Saudi Arabia; 4 Program of Physical Therapy, Department of Medical Rehabilitation Sciences, College of Applied Medical Sciences, King Khalid University, Abha, Saudi Arabia; 5 Department of Orthopedic Surgery, College of Medicine, King Khalid University, Abha, Saudi Arabia

**Keywords:** biomechanics, children, hypermobility, joint hypermobility, musculoskeletal pain, physical activity, Saudi Arabia, schoolbag

## Abstract

**Background:**

Musculoskeletal (MSK) pain is increasingly recognized as a cause of functional impairment in school-age children, yet its lifestyle and biomechanical correlates remain under-investigated in clinical populations.

**Objectives:**

To determine the 1-month prevalence and anatomical distribution of activity-limiting MSK pain among children aged 8–15 years and to identify lifestyle and biomechanical factors independently associated with its presence and impact.

**Methods:**

A cross-sectional study was conducted across multiple pediatric physiotherapy and orthopedic outpatient clinics in Saudi Arabia, involving 550 of 683 consecutively screened children (mean age: 11.45 ± 2.01 years; 50.55% male). Ethical approval was obtained from the Research Ethics Committee of King Khalid University (REC#234-2023). Data were collected on screen time, physical activity, backpack load, Beighton score (joint hypermobility), Foot Posture Index-6 (FPI-6), BMI, lower-limb strength, and Y-Balance test. Pain prevalence, intensity (NRS), anatomical distribution, and functional impact were assessed. Bivariate and multivariable analyses were conducted using SPSS v24. A *p*-value <0.05 was considered statistically significant.

**Results:**

The 1-month prevalence of activity-limiting MSK pain was 28.36%, with the back (39.74%), shoulders (30.77%), and knees (28.21%) being the most affected regions. Pain was more common in older children (12–15 years: 33.81%) and females (33.09%). Mean pain intensity was 5.93 ± 1.82. Children with pain had significantly higher screen time, backpack loads, Beighton scores, FPI-6, BMI, and lower-limb strength deficits (*p* < 0.05 for all). Logistic regression identified screen time > two h/day (AOR: 1.84), backpack load >10% (AOR: 2.12), hypermobility (AOR: 1.95), and reduced strength and balance as significant predictors. The interaction between high backpack load and hypermobility showed the strongest association (AOR: 2.87).

**Conclusion:**

Activity-limiting MSK pain affects nearly one-third of school-aged children and is strongly associated with modifiable lifestyle and biomechanical risk factors. Targeted screening and early interventions are warranted in pediatric settings.

## 1 Introduction

Musculoskeletal (MSK) pain is increasingly recognized as an important condition affecting children and adolescents, with consequences that go beyond immediate discomfort to include decreased physical activity, academic disruptions, and a long-term risk of developing chronic pain syndromes in adulthood (1). Although it was once thought to be uncommon in pediatric populations, recent research shows that MSK pain is quite common among school-aged children, especially during periods of rapid growth and increasing physical demands (2). The World Health Organization has identified childhood MSK health as a growing priority because of its potential to impact lifelong musculoskeletal health (3). In school and clinical settings, activity-limiting MSK pain can hinder children’s participation in age-appropriate activities such as walking, playing, carrying school supplies, and attending class, highlighting the importance of early detection and targeted intervention strategies (4).

Previous studies have reported wide-ranging prevalence estimates of pediatric MSK pain, typically between 20 and 40%, depending on the population sampled, definition of pain, and recall period used (5, 6). Anatomically, the back, knees, shoulders, and neck have consistently been identified as the most commonly affected regions (7). Notably, epidemiological data indicate that MSK pain in children is not a transient or benign phenomenon; it is associated with recurrence, psychosocial distress, and reduced health-related quality of life (8). Several factors have been proposed to influence the onset and severity of pain, including sedentary behavior, excessive screen time, heavy schoolbag loads, and low physical activity (9). In addition to modifiable behavioral factors, non-modifiable contributors such as congenital musculoskeletal anomalies, hereditary connective tissue disorders (e.g., hypermobility spectrum disorders), and familial pain sensitivity profiles have also been implicated in the etiology of pediatric MSK pain, potentially interacting with environmental stressors to amplify symptom expression (10–12). In addition, biomechanical characteristics such as joint hypermobility, altered foot posture, increased body mass index (BMI), impaired balance, and muscle weakness have been implicated in the etiology of pain episodes (13). However, findings across studies remain inconsistent, likely due to methodological variability and limited control for confounding variables.

Despite growing interest in pediatric MSK pain, few clinical investigations have simultaneously examined the interaction of modifiable lifestyle factors and intrinsic biomechanical characteristics in children presenting with activity-limiting symptoms (14). Most existing literature is either school-based or narrowly focused on a single risk factor, and it lacks comprehensive assessments using validated clinical tools (9). Furthermore, there is limited evidence derived from real-world pediatric physiotherapy or orthopaedic outpatient contexts, where children often seek care for functionally significant pain. Understanding the combined effects of daily behaviors (screen time, physical activity) and physical risk markers (Beighton score, foot posture, strength) is essential to identify children at the highest risk for pain-related disability (15, 16). This gap in integrated, clinic-based research limits the development of tailored prevention and rehabilitation strategies suitable for pediatric healthcare environments (16).

The present study was designed to address this need by investigating both the prevalence and functional impact of activity-limiting MSK pain in school-aged children attending a pediatric clinical center and by evaluating its association with a comprehensive set of lifestyle and biomechanical risk factors. Specifically, the objectives were (1) to determine the 1-month prevalence and anatomical distribution of activity-limiting MSK pain, and (2) to identify the independent lifestyle and biomechanical predictors of such pain. It was hypothesized that higher screen time, greater backpack loads, lower physical activity levels, joint hypermobility, abnormal foot posture, elevated BMI, reduced lower-limb strength, and impaired balance would be significantly associated with increased odds of activity-limiting MSK pain.

## 2 Methods

### 2.1 Study design, ethics, and settings

The present multi-centre cross-sectional study was conducted between June 2023 and April 2024 across four pediatric outpatient clinics located in different regions of Saudi Arabia: Abha, Riyadh, Jeddah, and Dammam. Reporting of this study follows the STROBE (Strengthening the Reporting of Observational Studies in Epidemiology) guidelines for cross-sectional studies. A completed checklist is provided in Supplementary file 1. Key demographic characteristics and data collection periods for each center are summarized in Supplementary Appendix Table A. Each site provided pediatric physiotherapy or musculoskeletal services and followed a standardized assessment protocol. Ethical approval was obtained from the Research Ethics Committee of King Khalid University [approval number: (REC#234-2023)], and all procedures were conducted in full compliance with the ethical standards outlined in the Declaration of Helsinki. This approval was formally secured before the initiation of participant recruitment and data collection at all study sites, in accordance with institutional and international ethical standards. Before participation, written informed consent was obtained from parents or legal guardians, and assent was obtained from all children in accordance with age-appropriate ethical guidelines.

### 2.2 Participants

Participants were consecutively recruited from pediatric physiotherapy outpatient departments at four regional clinics. Eligible children were aged 8 to 15 years and reported musculoskeletal pain within the past month that limited participation in daily or physical activities, as confirmed through structured clinical screening. Exclusion criteria included systemic inflammatory or neuromuscular disorders, recent fractures or surgeries, cognitive or developmental impairments affecting test performance, or ongoing treatment for chronic MSK conditions. A total of 683 children were screened, and 550 met inclusion criteria and were enrolled. Eligibility was verified through standardized clinical interviews, physical examinations, and baseline assessments conducted by licensed pediatric physiotherapists.

### 2.3 Primary outcome: activity-limiting musculoskeletal pain

The primary outcome was the presence of musculoskeletal (MSK) pain that limited engagement in age-appropriate physical activities within the previous 30 days. This was determined via structured, face-to-face interviews conducted jointly with the child and parent/guardian during the clinic visit. MSK pain was classified as “activity-limiting” if it disrupted participation in typical daily tasks, such as walking, stair climbing, recreational play, or carrying school materials, consistent with operational definitions (17). A binary outcome (Yes/No) was derived based on child and parental self-report, supported by physiotherapist confirmation. To assess the reliability of this binary classification, inter-rater agreement was evaluated in a subsample of 30 children at one site. Two physiotherapists independently applied the structured screening protocol during the same visit, blinded to each other’s ratings. Cohen’s *κ* was 0.84 (95% CI: 0.68–0.97), indicating strong inter-rater reliability for the primary outcome. Pain intensity was quantified using an 11-point Numeric Rating Scale (NRS), with endpoints of 0 (“no pain”) and 10 (“worst imaginable pain”). Children were instructed to rate their current pain or the most intense pain experienced in the past month. The location of pain was recorded using a standardized pediatric body map and categorized into anatomical regions: back, shoulder, knee, neck, ankle/foot, and other.

### 2.4 Functional impact variables

Functional limitations associated with MSK pain were evaluated across three domains: school absenteeism, sleep disturbance, and physical task performance (18). School absenteeism was recorded as the number of whole days missed due to pain in the preceding month, as reported by the parent/guardian. Sleep disturbance was assessed dichotomously (present/absent) through direct questioning, and its frequency was recorded in terms of the number of nights per week. Physical function was evaluated through NRS-based self-report ratings for specific tasks: walking, stair climbing, playing, and carrying a school bag. Each activity was rated on a scale from 0 (“no difficulty”) to 10 (“unable to perform”), adapted. These outcomes were treated as continuous variables and analyzed only among participants who reported activity-limiting pain.

### 2.5 Lifestyle exposure variables

Screen time was measured in hours per day using a structured questionnaire based on child and parent recall, referencing a typical weekday and weekend, and averaged accordingly. Physical activity was quantified as the cumulative weekly duration (hours/week) of structured and unstructured physical activities, including sports and outdoor play, using open-ended recall protocols recommended by the WHO Global Physical Activity Questionnaire (GPAQ, adapted for children) (18). Although the GPAQ adaptation is widely used in child health surveillance, its reliability may be limited in pediatric populations due to variable recall accuracy, particularly among younger children. To evaluate the reliability of this tool in the current context, a test–retest analysis was conducted in a subsample of 36 participants at the Abha clinic, with a 7-day interval between assessments. The intraclass correlation coefficient (ICC) for total weekly physical activity was 0.82 (95% CI: 0.70–0.90), reflecting good test–retest reliability for the adapted GPAQ-child under field conditions (19). Backpack load was measured directly at the clinic using a digital portable scale (±0.1 kg precision). Children were instructed to wear their usual school backpack on arrival; the bag weight was divided by the child’s body weight (measured with a calibrated digital scale) and expressed as a percentage. To ensure ecological validity, participants were specifically asked to bring the same backpack and contents they typically carry to school, reflecting their usual daily load. A threshold of greater than 10% body weight was used to define an excessive backpack load, following ergonomic recommendations (20).

### 2.6 Biomechanical and anthropometric variables

Generalized joint hypermobility was assessed using the Beighton Score (range: 0–9), a validated tool comprising five bilateral maneuvers and one unilateral spinal flexion test. A score ≥4 was used to define hypermobility in children, consistent with pediatric population norms. Foot posture was evaluated using the Foot Posture Index-6 (FPI-6) (21), which assesses six clinical criteria (e.g., talar head palpation, arch congruence), each scored from −2 to +2, resulting in a total score ranging from −12 to +12. Higher scores indicated greater pronation. Body mass index (BMI) was calculated as weight in kilograms divided by height in meters squared (kg/m^2^), and children were categorized as underweight, normal, overweight, or obese according to the WHO 2007 growth reference percentiles adjusted for age and sex.

### 2.7 Physical performance variables

Lower-limb muscle strength was assessed using a handheld dynamometer (MicroFET2, Hoggan Scientific) during maximal isometric quadriceps contraction (22). The test was performed on the dominant leg with the child seated, knee at 90° flexion, and stabilization provided by the examiner. Three trials were conducted with 30-s rest intervals, and the highest value (in Newtons) was retained for analysis. Dynamic balance was measured using the Y-Balance Test, a reliable and validated assessment adapted for pediatric use (23). Reach distances in the anterior, posteromedial, and posterolateral directions were recorded, and a composite score was calculated as the average of the three directions, normalized to leg length (% limb length). Lower scores indicated poorer neuromuscular control.

All measurements were performed by trained pediatric physiotherapists who underwent standardized calibration and inter-rater reliability checks before data collection, ensuring consistency and measurement validity. To quantify inter-rater agreement, a reliability subsample of 32 children was independently assessed by two physiotherapists at each site using identical protocols. Intraclass correlation coefficients demonstrated high reliability across tools: Beighton score (ICC = 0.91), FPI-6 (ICC = 0.88) (24), handheld dynamometry (ICC = 0.93) (25), and Y-Balance composite score (ICC = 0.89) (26), confirming consistent measurements across raters and settings.

### 2.8 Sample size estimation

The sample size was calculated using G*Power based on a hypothesized link between backpack loads >10% of body weight and musculoskeletal pain, with assumed pain prevalence of 25% (unexposed) vs. 40% (exposed) informed by internal clinic audits. Using an alpha of 0.05, power of 0.90, and an odds ratio of 2.0, the minimum required sample was 420. This was increased to 550 to allow for multivariable modeling and potential missing data. A sensitivity analysis showed that detecting smaller effect sizes (OR = 1.5) would require 700 participants, while larger effects (OR = 2.5) would need 300, confirming sufficient power to detect moderate-to-strong associations.

### 2.9 Data analysis

All statistical analyses were performed using IBM SPSS Statistics version 24.0 (IBM Corp., Armonk, NY, USA). Continuous variables were summarized as means with standard deviations, while categorical variables were summarized as frequencies with percentages. Normality was confirmed through visual inspection of histograms and the Shapiro–Wilk test, allowing for the use of parametric tests. Between-group comparisons were conducted using independent samples *t*-tests for continuous variables and chi-square tests for categorical variables. Functional impact measures [e.g., Numeric Rating Scale (NRS) scores for walking, stair climbing, play, and sleep disturbance] were analyzed descriptively and compared using the same methods. To identify independent predictors of activity-limiting musculoskeletal pain (binary outcome: yes/no), a multivariable logistic regression analysis was performed. Covariates included key lifestyle factors (screen time, backpack load, physical activity) and biomechanical measures (BMI, Beighton score, foot posture, lower-limb strength, dynamic balance). Variables with *p* < 0.10 in bivariate analysis were entered into the multivariable model using the enter method. Since the bivariate analyses were exploratory, no formal correction for multiple comparisons was applied; however, the risk of Type I error was addressed through multivariable logistic regression, where only variables showing independent associations were kept and interpreted. Interaction terms were tested to explore potential synergistic effects (e.g., excessive backpack load × hypermobility). Multicollinearity was assessed using Variance Inflation Factors (VIF) for all covariates in the final model. All VIF values were below 2.5 (range: 1.12–2.47), indicating low intercorrelation and supporting the retention of all variables. Model fit was evaluated with the Hosmer–Lemeshow goodness-of-fit test, and predictive ability was assessed using Nagelkerke R^2^ and the area under the receiver operating characteristic curve (AUC). A two-tailed *p*-value <0.05 was considered statistically significant for all analyses. All variables had less than 5% missing data; thus, a complete-case analysis was performed without the need for imputation. Data quality was maintained through standardized electronic entry procedures and real-time validation at each study site.

## 3 Results

The participant group (*N* = 550) had a balanced gender distribution and an average age of 11.45 years. Most (64.00%) were within the normal BMI range, while 28.37% were overweight or obese, reflecting a typical pediatric clinical sample (Table 1). The average screen time was over three hours daily, and physical activity was modest, just under five hours per week. Backpack weights averaged 12.23% of body weight, exceeding the commonly recommended threshold and potentially contributing to mechanical stress. The mean Beighton score was 3.12, indicating a significant proportion with joint hypermobility. Foot posture was moderately pronated. Physical performance measures showed a mean lower-limb strength of 142.68 N and a Y-Balance composite score of 83.45 cm, suggesting variability in strength and balance within the sample.

**Table 1:** Participant characteristics.

Variable	Mean ± SD or *n* (%)
Age (years)	11.45 ± 2.01
Sex (Male)	278 (50.55%)
BMI (kg/m^2^)	19.82 ± 3.45
BMI category - underweight	42 (7.63%)
BMI category - normal	352 (64.00%)
BMI category - overweight	98 (17.82%)
BMI category - obese	58 (10.55%)
School grade (Mean ± SD)	5.6 ± 1.3
Screen time (hours/day)	3.72 ± 1.48
Physical activity (hours/week)	4.93 ± 2.15
Backpack load (% body weight)	12.23 ± 3.41
Beighton score (0–9)	3.12 ± 2.35
Foot posture index (FPI-6)	5.34 ± 2.02
Y-balance composite score (cm)	83.45 ± 10.87
Lower-limb strength (N)	142.68 ± 29.44

BMI, body mass index; FPI-6, foot posture index-6; SD, standard deviation; N, Newtons.

**Figure 1:**
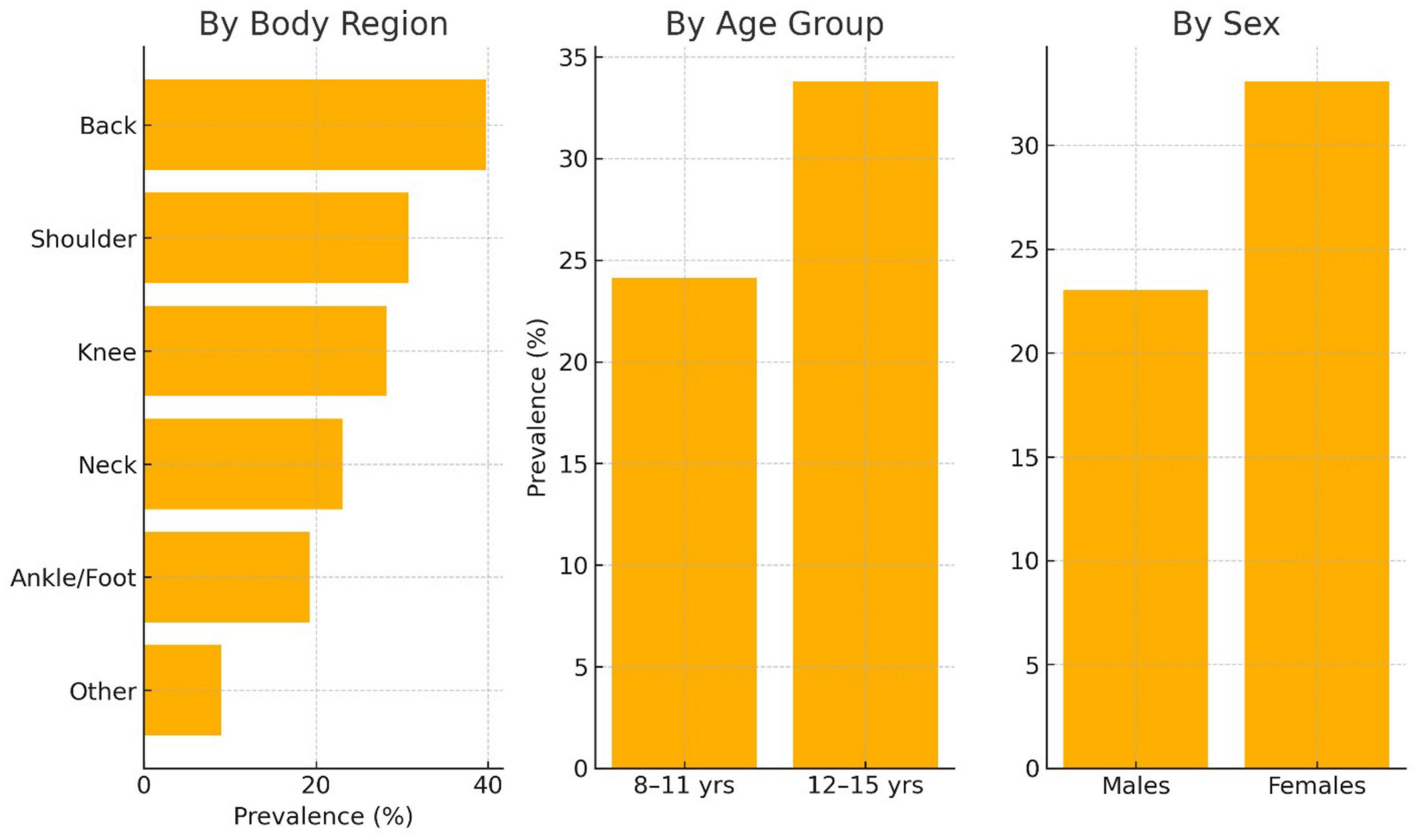
Distribution of activity-limiting musculoskeletal pain by body region, age group, and sex.

Activity-limiting musculoskeletal pain affected 28.36% of school-aged children in the past month, with the back being the most commonly reported pain site, followed by the shoulders and knees (Table 2; Figure 1). Pain was more common among older children (12–15 years: 33.81%) compared to younger ones (8–11 years: 24.12%), and was significantly higher in females (33.09%) than males (23.02%). The average pain intensity, measured with a numeric rating scale, was moderate (mean NRS: 5.93 ± 1.82), indicating clinically relevant discomfort that could affect daily functioning.

**Table 2:** Prevalence and anatomical pattern of activity-limiting musculoskeletal pain.

Variable	*n* (%) or mean ± SD
1-month prevalence of activity-limiting pain	156 (28.36%)
Back pain	62 (39.74%)
Shoulder pain	48 (30.77%)
Knee pain	44 (28.21%)
Neck pain	36 (23.08%)
Ankle/foot pain	30 (19.23%)
Other regions	14 (8.97%)
Mean pain intensity (NRS, 0–10)	5.93 ± 1.82
Pain prevalence in 8–11 yrs	62 (24.12%)
Pain prevalence in 12–15 yrs	94 (33.81%)
Pain prevalence in males	64 (23.02%)
Pain prevalence in females	92 (33.09%)

NRS, numeric rating scale; SD, standard deviation.

Among children experiencing activity-limiting musculoskeletal pain, notable functional impairments were observed across multiple areas, with an average of 2.38 school days missed in the past month. Over half of the affected children (53.85%) reported sleep disturbances (Table 3). Sleep disruption happened about three nights per week on average, indicating a significant impact on rest and recovery. Pain-related limitations in daily activities were moderate, with the greatest difficulty reported during play (mean NRS: 5.43), followed by carrying school bags (4.79), climbing stairs (4.86), and walking (4.12), all reflecting meaningful interference with age-appropriate physical function.

**Table 3:** Functional impact of pain among affected children.

Variable	Mean ± SD or *N* (%)	95% CI
School days missed (past month)	2.38 ± 1.72	2.10–2.66
Sleep disturbance (Yes)	84 (53.85%)	—
Sleep disturbance frequency (nights/week)	2.96 ± 1.21	2.71–3.21
Difficulty walking (NRS 0–10)	4.12 ± 2.34	3.79–4.45
Difficulty climbing stairs (NRS 0–10)	4.86 ± 2.17	4.56–5.16
Difficulty playing (NRS 0–10)	5.43 ± 2.61	5.07–5.79
Difficulty carrying school bag (NRS 0–10)	4.79 ± 2.45	4.49–5.09

NRS, numeric rating scale; SD, standard deviation; CI, confidence interval; *N*, number of participants.

**Figure 2:**
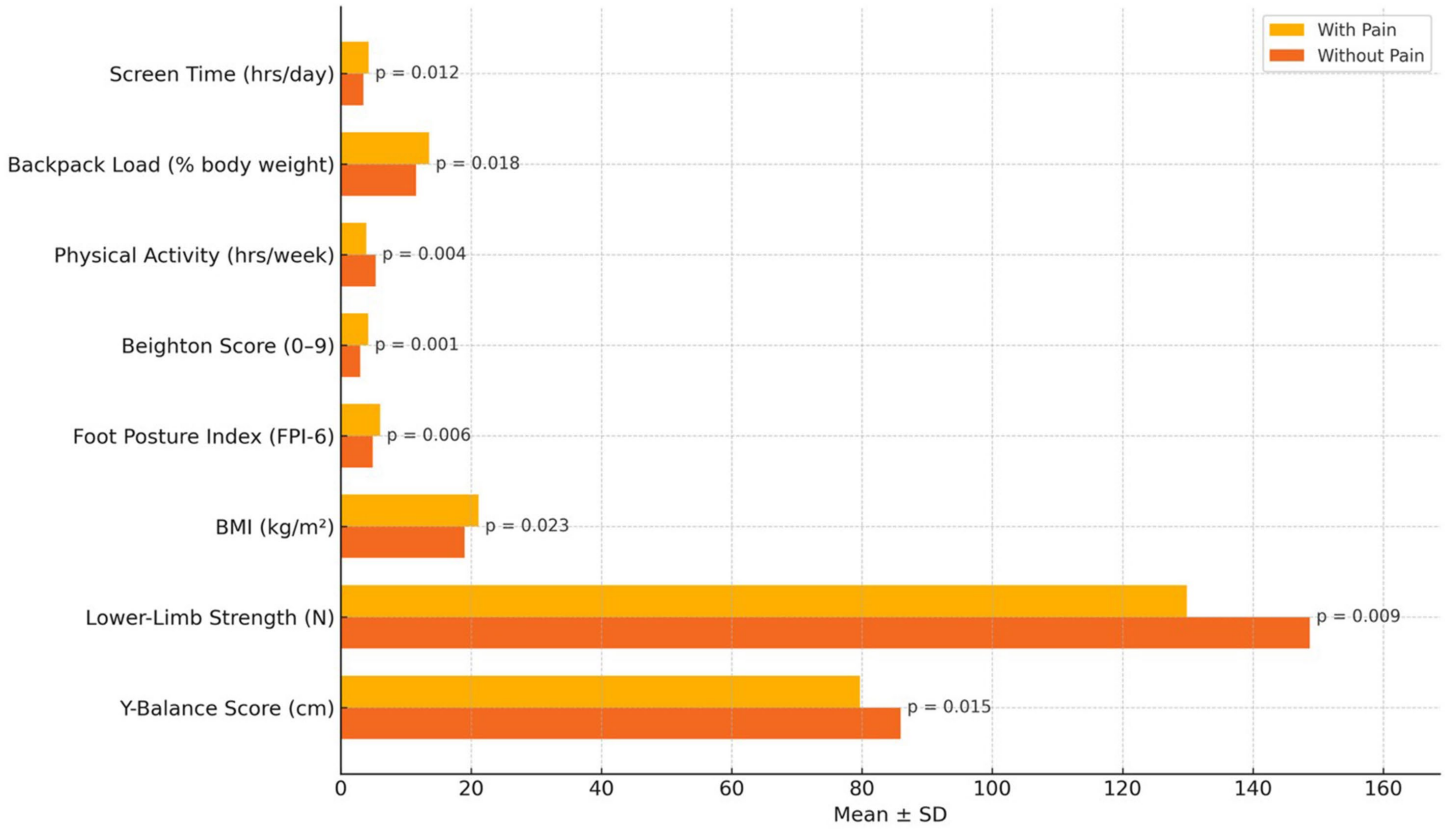
Comparison of lifestyle and biomechanical factors between children with and without activity-limiting pain.

Children with activity-limiting musculoskeletal pain showed significantly less favorable profiles across various lifestyle and biomechanical factors compared to their pain-free peers (Table 4; Figure 2). Notably, those with pain reported more screen time and backpack loads, along with lower physical activity levels (*p* < 0.05 for all). Biomechanically, children affected by pain showed greater joint hypermobility, more pronated foot posture, higher BMI, and reduced lower-limb strength and dynamic balance, all of which had statistically significant differences (*p*-values from 0.001 to 0.023). These findings suggest that both modifiable lifestyle behaviors and inherent physical factors may play a role in pain-related disability in this group.

**Table 4:** Bivariate associations between pain and lifestyle/biomechanical factors.

Variable	With pain (Mean ± SD)	Without pain (Mean ± SD)	*p*-value
Screen time (hrs/day)	4.26 ± 1.39	3.47 ± 1.32	0.012
Backpack load (% body weight)	13.54 ± 2.98	11.54 ± 2.71	0.018
Physical activity (hrs/week)	3.91 ± 1.75	5.34 ± 2.01	0.004
Beighton score (0–9)	4.23 ± 2.08	2.98 ± 1.86	0.001
Foot posture index (FPI-6)	6.02 ± 1.83	4.87 ± 1.71	0.006
BMI (kg/m^2^)	21.11 ± 3.24	19.03 ± 3.18	0.023
Lower-limb strength (N)	129.84 ± 26.45	148.72 ± 28.56	0.009
Y-balance composite score (cm)	79.65 ± 11.07	85.93 ± 10.14	0.015

BMI, body mass index; FPI-6, foot posture index-6; N, Newtons; SD, standard deviation.

**Figure 3:**
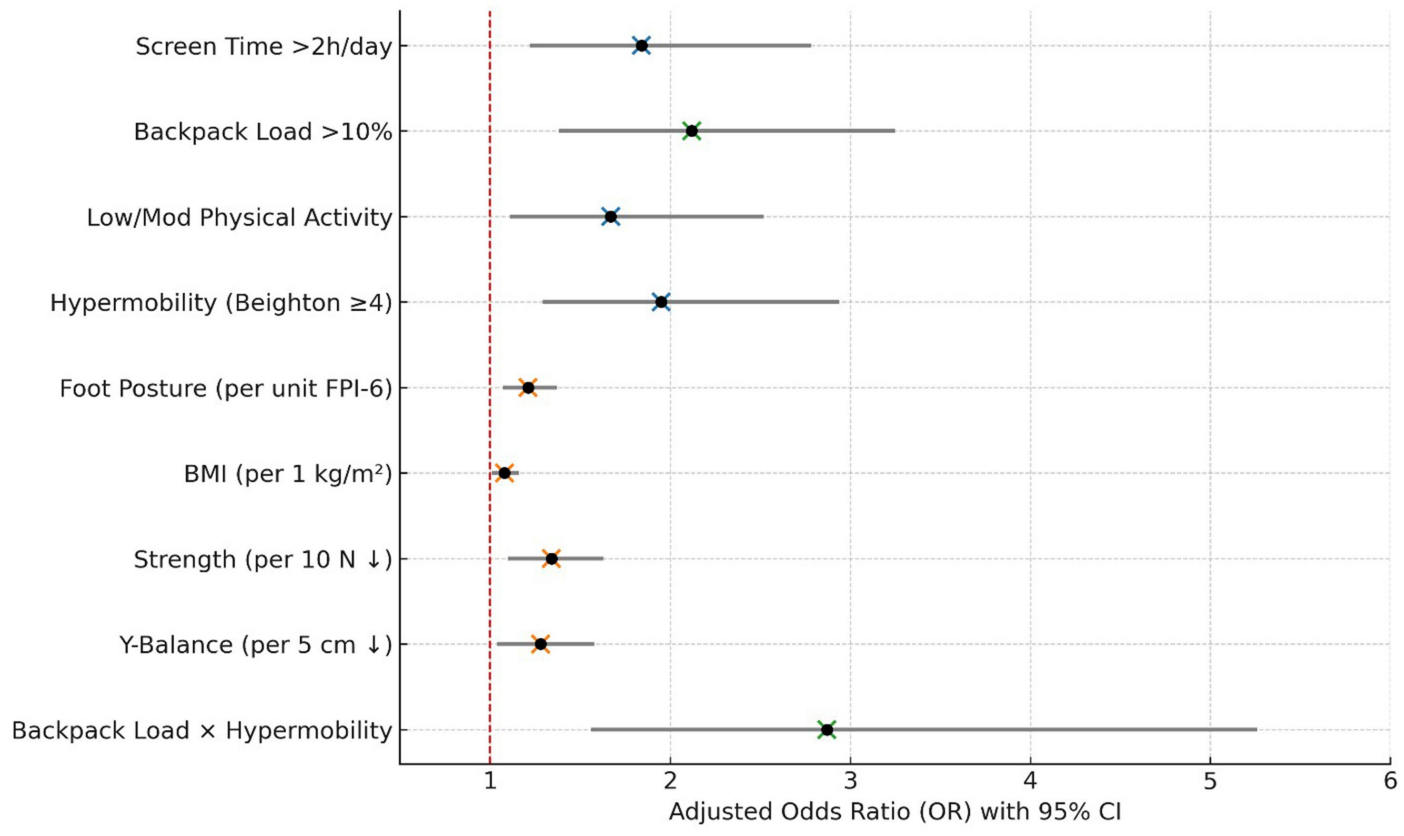
Multivariable logistic regression: predictors of activity-limiting musculoskeletal pain with 95% confidence intervals.

**Figure 4:**
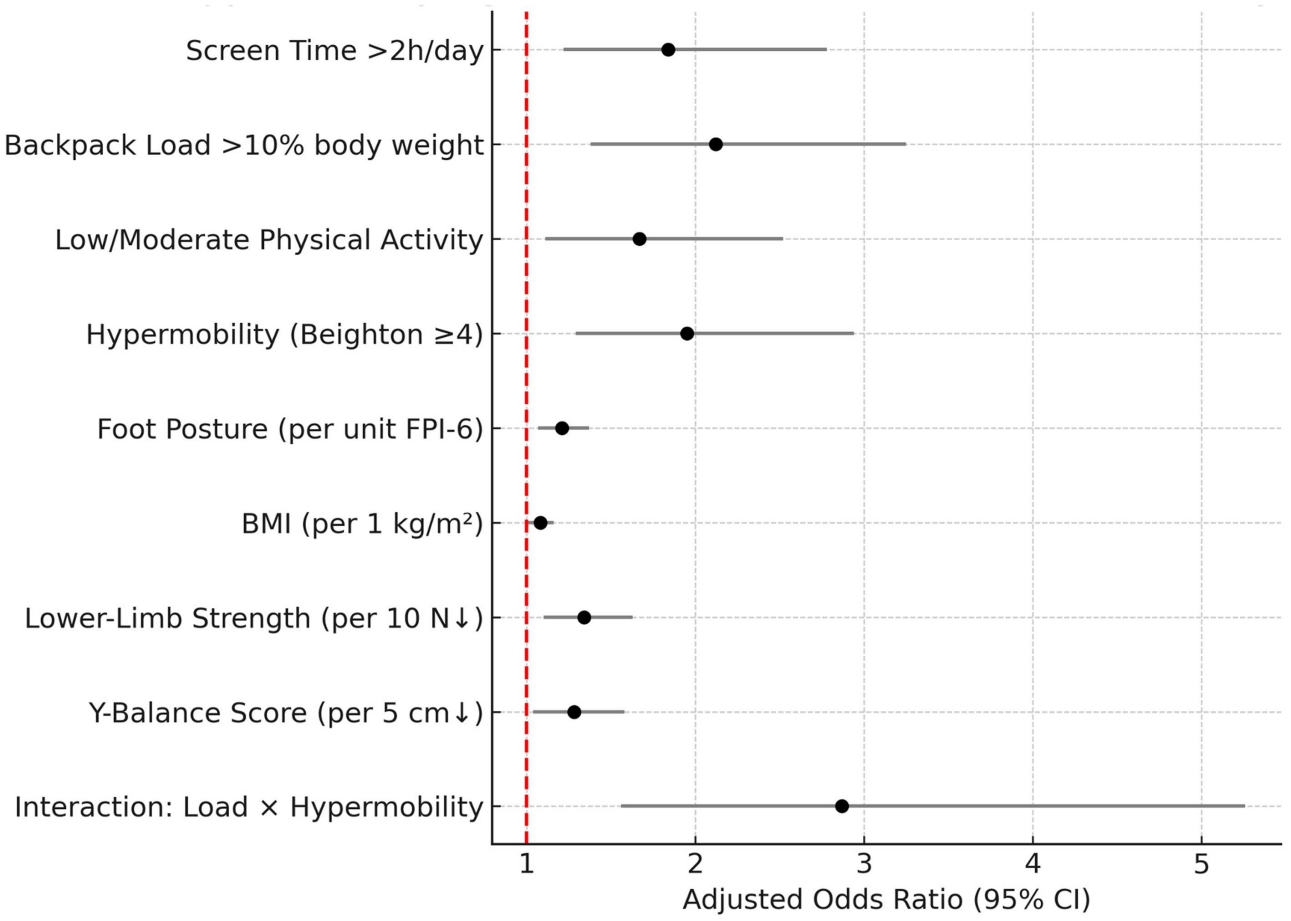
Forest plot displaying adjusted odds ratios and 95% confidence intervals for all predictors of activity-limiting musculoskeletal pain. Continuous variables are scaled according to the units used in the logistic regression model: per unit increase in FPI-6, per 1 kg/m*^2^* increase in BMI, per 10 N decrease in lower-limb strength, and per 5 cm decrease in Y-Balance score.

In multivariable logistic regression, several lifestyle and biomechanical factors emerged as significant independent predictors of activity-limiting musculoskeletal pain (Figures 3, 4). Children exceeding two hours of daily screen time (AOR: 1.84), carrying backpack loads >10% of body weight (AOR: 2.12), engaging in low/moderate physical activity (AOR: 1.67), and exhibiting generalized joint hypermobility (AOR: 1.95) were all at increased risk. Additionally, higher foot posture index and BMI, along with reduced lower-limb strength and Y-Balance scores, were associated with increased odds, albeit with milder effect sizes. Notably, the interaction between excessive backpack load and hypermobility demonstrated the most potent effect (AOR: 2.87), suggesting a synergistic risk. Stratified analyses revealed that hypermobility (Beighton score ≥ 4) was associated with pain in both backpack load subgroups, but the association was significantly stronger among children carrying >10% body weight. The odds ratio for hypermobility was 1.42 (95% CI: 0.81–2.49, *p* = 0.22) in the ≤10% group and 2.61 (95% CI: 1.52–4.47, *p* = 0.001) in the >10% group. The interaction term was statistically significant (p-for-interaction = 0.036), indicating effect modification by mechanical load. Model diagnostics indicated good fit (Hosmer-Lemeshow *p* = 0.411) and acceptable discriminative ability (AUC = 0.792). Observed-versus-expected frequencies by decile of predicted probability are shown in Supplementary Table S1, and a corresponding calibration plot is provided in Supplementary Figure S1. The close alignment between predicted and actual values across deciles further supports model calibration.

## 4 Discussion

This cross-sectional study aimed to examine the prevalence, anatomical distribution, and functional effects of activity-limiting musculoskeletal pain in school-aged children, as well as to explore its links with key lifestyle and biomechanical factors. The results showed a significant burden of pain, mainly affecting the axial and lower limb regions, with notable differences in prevalence by age and sex. Pain was linked to important functional limitations, such as missed school days, sleep disturbances, and difficulty with daily activities. Bivariate and multivariable analyses identified several modifiable and intrinsic factors that were statistically associated with pain; however, due to the cross-sectional design, these associations do not imply causation or confirm the temporal direction of relationships.

The observed prevalence of activity-limiting musculoskeletal pain in this study (28.36%) aligns with previous reports showing rates between 20 and 40% in school-aged populations, reinforcing the significant burden of MSK pain during late childhood and adolescence (7, 22). However, it is important to contextualize this figure within the nature of the sample, which was drawn exclusively from outpatient physiotherapy and orthopedic clinics (27). As such, the prevalence estimate likely reflects a higher-risk, symptom-presenting population rather than the general pediatric population. This referral-based sampling approach may overestimate prevalence compared to community-based studies and should be interpreted accordingly. Notably, the most affected areas—back (39.74%), shoulders (30.77%), and knees (28.21%)—correspond with regions of increased mechanical demand and align with previous findings in similar age groups (28). The higher prevalence among older children (12–15 years: 33.81%) and females (33.09%) is consistent with known developmental and sex-related differences in musculoskeletal sensitivity and pain reporting (29).

Recent school-based studies conducted in the Middle East have reported lower prevalence rates of musculoskeletal pain compared to the 28.36% observed in this clinical sample (30). Daajani et al. (31) found a prevalence of 18.5% among Saudi adolescents aged 12–16 years, while a cross-sectional study by Dawod et al. (32) in Jordan reported rates of 21.3% in a similar age group (32). A 2023 UAE-based study by Baniissa et al. (33) noted a prevalence of 16.7% among schoolchildren aged 10–14. These differences likely reflect the referral bias inherent in clinic-based recruitment, where children presenting for care are more likely to have functionally limiting or persistent symptoms (32). Thus, while the present findings fall within the broader regional range, the elevated prevalence highlights the burden among help-seeking populations and reinforces the need for early detection and targeted intervention in primary care and school health systems.

The observed links between activity-limiting musculoskeletal pain and lifestyle and biomechanical factors can be explained by existing evidence that highlights the complex origins of pediatric pain (34). Excessive screen time and decreased physical activity are linked to musculoskeletal problems through prolonged sitting and poor posture habits, as reported by Chen et al. (35) and de Souza et al. (9). Additionally, carrying school bags that weigh more than 10% of a child’s body weight puts repetitive stress on the spine, with earlier studies by Feldman et al. (36) and León-Domínguez et al. (37) showing higher rates of back and shoulder pain in such cases. Joint hypermobility, a known internal risk factor, has been associated with altered neuromuscular control and mechanical instability (11), consistent with findings by Hart et al. (11) and Ituen et al. (12). Increased foot posture index and BMI also impact weight distribution during movement and are linked to pain in children’s lower limbs (38, 39). The reduced lower-limb strength and balance observed in this study support the idea that impaired musculoskeletal performance contributes to pain development (12). Importantly, the increased risk associated with the combination of heavy backpack loads and hypermobility underscores the complex interaction between modifiable and structural factors, emphasizing the importance of targeted prevention for vulnerable pediatric groups (40).

The functional effects of activity-limiting musculoskeletal pain in children were evident across academic, sleep, and physical activity domains, aligning with previous pediatric pain research (35, 36). School absences and disrupted sleep patterns have been previously identified as significant outcomes of chronic pain in children, with studies by Solé et al. (37) and Miró et al. (25) highlighting the cumulative impact on learning, mood, and overall well-being. The reported difficulties in basic mobility and participation in play match findings by Seyhan-Bıyık et al. (25), who observed how ongoing musculoskeletal pain affects motor performance and physical independence in school-aged children. The higher difficulty scores for climbing stairs, walking, and carrying school bags indicate challenges with routine weight-bearing activities, likely worsened by biomechanical stressors such as joint hypermobility and decreased muscle strength, as described by Lamari et al. (41). These findings reinforce the idea that musculoskeletal pain in children, even at moderate levels, has broad and tangible effects on function, emphasizing the importance of early recognition and intervention (12).

This study has several methodological strengths. It represents the first clinic-based, multi-region investigation of musculoskeletal pain in Saudi school-aged children that concurrently evaluates validated biomechanical assessments and lifestyle exposures. The large sample size (*N* = 550) and *a priori* power calculation ensured sufficient statistical precision for the primary hypothesis. The inclusion of objective physical performance measures, such as handheld dynamometry and the Y-Balance test, enhanced construct validity by minimizing reliance on subjective self-report. Furthermore, the use of multi-domain functional impact outcomes—including school absenteeism, sleep disturbance, and task-specific physical limitations—added clinically meaningful context to the prevalence and risk factor findings.

### 4.1 Clinical significance

This study offers clinically meaningful insights into the multifactorial nature of activity-limiting musculoskeletal pain in school-aged children, highlighting both modifiable lifestyle behaviors and inherent biomechanical traits as key contributors. Identifying excessive screen time, heavy backpack loads, and insufficient physical activity as independent risk factors underscores the need for targeted, behavior-based interventions in school and community settings. Furthermore, the links with generalized joint hypermobility, altered foot posture, higher BMI, and deficits in lower-limb strength and balance highlight the importance of personalized musculoskeletal screening during pediatric health checkups. The observed functional impairments—including decreased school attendance, disrupted sleep, and difficulty performing routine physical tasks—further underscore the clinical burden of musculoskeletal pain in this age group. Notably, the combined effect of high mechanical load and hypermobility as a strong predictor of pain risk suggests that multidisciplinary strategies that include education, ergonomic adjustments, physical therapy, and ongoing clinical monitoring may be necessary for effective prevention and early treatment.

Maintaining schoolbag loads within the recommended 10% of body weight presents practical challenges in the Saudi educational context. Structural factors such as double-shift schedules, lack of locker infrastructure, and the need to carry multiple textbooks daily contribute to consistently elevated carriage loads. Although the 10% threshold originates from North American guidelines established in the 1990s, its application requires contextual adaptation (42). Feasible mitigation strategies include installing secure storage options (lockers or cubbies) and implementing digital or modular scheduling systems to reduce daily material volume. Both approaches have demonstrated efficacy in reducing mechanical strain and improving musculoskeletal outcomes in school-aged children and should inform future ergonomic and educational policies. While the study identifies associations between backpack load and pain, interpretations must be cautious due to the cross-sectional design, which limits causal inference. To enhance clinical applicability, ROC-derived thresholds were calculated for key physical performance measures. A quadriceps strength cut-off of <132 N identified children with activity-limiting pain with 76% sensitivity and 68% specificity, while a Y-Balance composite score <81 cm yielded 72% sensitivity and 65% specificity. These preliminary thresholds may support screening efforts but require validation in prospective pediatric cohorts.

### 4.2 Limitations and future directions

This study has several methodological limitations. The cross-sectional design limits causal inference, preventing conclusions about the directionality of observed associations. Recruitment from outpatient physiotherapy and orthopedic clinics may have introduced selection bias, as children presenting for care are more likely to exhibit severe or functionally limiting symptoms, potentially inflating prevalence estimates and limiting generalizability to community populations. Self- or parent-reported measures of screen time and physical activity are susceptible to recall and social desirability bias, particularly in younger children, due to the limited reliability of the adapted WHO GPAQ. Similarly, backpack weight was measured on a single clinic day and may not reflect peak mid-week loads, reducing ecological precision. Although all assessments were conducted by trained physiotherapists using validated tools, some inter-rater variability may have affected foot posture and muscle strength measurements. From a statistical perspective, the inclusion of ten predictors for 156 outcome events approaches the conventional events-per-variable threshold, posing a minor risk of overfitting. Continuous covariates such as BMI, FPI-6, quadriceps strength, and Y-Balance scores were modeled as linear predictors without formal checks for non-linearity (e.g., spline or fractional polynomial transformations). Multiple comparisons in the bivariate analysis were not adjusted for, as these were exploratory and intended to inform the multivariable model. Although minimal clinically important differences (MCIDs) were not integrated into the primary analysis, pediatric literature suggests that changes of approximately 20 N in quadriceps strength and 4–5 cm in Y-Balance composite scores may correspond to meaningful shifts in pain or functional outcomes, supporting the interpretability of the observed effect sizes. Lastly, several potentially relevant confounders were not assessed, including socio-economic status, parental pain history, school furniture ergonomics, and academic workload. Future longitudinal and school-based studies should incorporate these variables to provide a more comprehensive understanding of pediatric musculoskeletal pain and inform targeted prevention strategies.

## 5 Conclusion

This study revealed that nearly one-third of school-aged children experienced activity-limiting musculoskeletal pain within a month, most commonly affecting the back, shoulder, and knee areas. Pain was more prevalent among older children and females and was associated with moderate difficulties in academics, sleep, and physical activity. Both bivariate and multivariable analyses identified significant connections between pain and lifestyle factors, such as screen time, backpack weight, and physical activity, as well as biomechanical factors, including joint hypermobility, foot posture, BMI, lower-limb strength, and dynamic balance. The combination of excessive mechanical load and generalized hypermobility emerged as a particularly strong predictor of pain. These findings underscore the importance of assessing both behavioral and physical risk factors in clinical and school settings to better understand the impact of musculoskeletal pain in children.

## Data Availability

The datasets presented in this study can be found in online repositories. The names of the repository/repositories and accession number(s) can be found in the article/Supplementary material.

## Supplementary material

SUPPLEMENTARY FIGURE S1 Calibration plot showing agreement between predicted and observed probabilities across Hosmer–Lemeshow deciles. The dashed line represents perfect calibration (predicted = observed).

## References

[1] Rinaldi JT Patel BT Ackerman RS. "Nutrition, obesity, and musculoskeletal pain". In: Musculoskeletal pain: Evidence-based clinical evaluation and management. Cham, Switzerland: Springer (2025). 51–75.

[2] Faienza MF Urbano F Chiarito M Lassandro G Giordano P. Musculoskeletal health in children and adolescents. Front Pediatr. (2023) 11:1226524. doi: 10.3389/fped.2023.1226524, 38161439 PMC10754974

[3] Paskins Z Farmer CE Manning F Andersson DA Barlow T Bishop FL . Research priorities to reduce the impact of musculoskeletal disorders: a priority setting exercise with the child health and nutrition research initiative method. Lancet Rheumatology. (2022) 4:e635–45. doi: 10.1016/S2665-9913(22)00136-9, 36275038 PMC9584828

[4] Johansson F Billquist J Andreasson H Jensen I Onell C Berman AH . Study environment and the incidence of mental health problems and activity-limiting musculoskeletal problems among university students: the SUN cohort study. BMJ Open. (2023) 13:e072178. doi: 10.1136/bmjopen-2023-072178 PMC10503358 37709330

[5] Stevens B.J. Zempsky W.T. Prevalence and distribution of pain in children. Oxford textbook of pediatric pain 2. Oxford, United Kingdom: Oxford University Press (2021).

[6] Thomas MJ Dunn KM. "Musculoskeletal conditions". In: Handbook of epidemiology. Cham, Switzerland: Springer (2024). 1–59.

[7] Rybski MF Juckett L. "Posture" In: Kinesiology for occupational therapy. New York, NY, USA: Routledge (2024). 261–89.

[8] Palermo T.M. Gorbounova I. Chronic and recurrent pain: Considerations for child health and well-being. Oxford, United Kingdom: Oxford University Press (2025).

[9] de Souza JM Tebar WR Delfino LD Tebar FS Gobbo LA Franco M . Association of musculoskeletal pain with sedentary behavior in public school teachers: the role of habitual physical activity. Pain Manag Nurs. (2023) 24:196–200. doi: 10.1016/j.pmn.2022.08.005, 36100514

[10] Lamari N Beighton P. Hypermobility in medical practice. Cham, Switzerland: Springer (2023).

[11] Romeo DM Venezia I De Biase M Ascione F Lala MR Arcangeli V . Developmental coordination disorder and joint hypermobility in childhood: a narrative review. Children. (2022) 9:1011. doi: 10.3390/children9071011 35883995 PMC9317025

[12] Ituen OA Duysens J Ferguson G Smits-Engelsman B. The strength of balance: strength and dynamic balance in children with and without hypermobility. PLoS One. (2024) 19:e0302218. doi: 10.1371/journal.pone.0302218 38923950 PMC11206839

[13] Lamari N Beighton P. "Mechanical consequences of joint hypermobility". In: Hypermobility in medical practice. Cham, Switzerland: Springer (2023). 63–71.

[14] Sadiasa A Werkmeister JA Gurung S Gargett CE. Steps towards the clinical application of endometrial and menstrual fluid mesenchymal stem cells for the treatment of gynecological disorders. Expert Opin Biol Ther. (2025) 25:285–307. doi: 10.1080/14712598.2025.2465826, 39925343

[15] Schubert Hjalmarsson E. Exploring pain, fatigue and physical activity in adolescents with hypermobility Spectrum disorder or hypermobile Ehlers-Danlos syndrome. Stockholm, Sweden: Karolinska Institutet (2024).

[16] Klein S Chiu K Clinch J Liossi C. "Musculoskeletal pain in children and young people". In: Managing pain in children and young people: A clinical guide. Cham, Switzerland: Springer (2024). 147–69.

[17] Turk DC Patel KV. "Epidemiology and economics of chronic and recurrent pain". In: Clinical pain management: A practical guide. Oxford, United Kingdom: Oxford University Press (2022). 6–24.

[18] Sabet TS Anderson DB Stubbs PW Buchbinder R Terwee CB Chiarotto A . Pain and physical function are common core domains across 40 core outcome sets of musculoskeletal conditions: a systematic review. J Clin Epidemiol. (2025) 180:111687. doi: 10.1016/j.jclinepi.2025.111687, 39864671

[19] Larouche R Abadi MRH Aubert S Bhawra J Brazo-Sayavera J Carson V . Development and validation of the global adolescent and child physical activity questionnaire (GAC-PAQ) in 14 countries: study protocol. BMJ Open. (2024) 14:e082275. doi: 10.1136/bmjopen-2023-082275, 39053955 PMC11284885

[20] Oulvan S. Yudasmara D.S. Sari Z.N. Winarno M. Fadhli N.R. Hariadi I., The association physical activity (3–5 years) to gross and fine motor ability of preschool children’s, 5th international scientific meeting on public health and sports (ISMOPHS 2023). Paris, France: Atlantis Press (2023) pp. 115–128.

[21] Hegazy F Aboelnasr E Abuzaid M Kim I-J Salem Y. Comparing validity and diagnostic accuracy of clarke’s angle and foot posture index-6 to determine flexible flatfoot in adolescents: a cross-sectional investigation. J Multidiscip Healthc. (2021) 14:2705–17. doi: 10.2147/JMDH.S317439, 34611407 PMC8486009

[22] Du W Cornett KM Donlevy GA Burns J McKay MJ. Variability between different hand-held dynamometers for measuring muscle strength. Sensors. (2024) 24:1861. doi: 10.3390/s24061861, 38544123 PMC10974287

[23] Plisky P Schwartkopf-Phifer K Huebner B Garner MB Bullock G. Systematic review and meta-analysis of the Y-balance test lower quarter: reliability, discriminant validity, and predictive validity. Int J Sports Phys Ther. (2021) 16:1190. doi: 10.26603/001c.27634 34631241 PMC8486397

[24] Evans AM Rome K Peet L. The foot posture index, ankle lunge test, Beighton scale and the lower limb assessment score in healthy children: a reliability study. J Foot Ankle Res. (2012) 5:1. doi: 10.1186/1757-1146-5-1, 22230105 PMC3283490

[25] Chamorro C Armijo-Olivo S la De Fuente C Fuentes J Javier Chirosa L. Absolute reliability and concurrent validity of hand held dynamometry and isokinetic dynamometry in the hip, knee and ankle joint: systematic review and meta-analysis. Open Med. (2017) 12:359–75. doi: 10.1515/med-2017-0052, 29071305 PMC5651404

[26] Zheng Y Feng R Hu W Huang P. Investigation of inter-rater and test-retest reliability of Y balance test in college students with flexible flatfoot. BMC Sports Sci Med Rehabil. (2024) 16:40. doi: 10.1186/s13102-024-00819-3, 38331956 PMC10854180

[27] Agyei-Mensah Y.O., Association of sedentary behavior with age and gender in Finnish school-age children. Kuopio, Finland: University of Eastern Finland (2024).

[28] Hébert JJ Beynon AM Jones BL Wang C Shrier I Hartvigsen J . Spinal pain in childhood: prevalence, trajectories, and diagnoses in children 6 to 17 years of age. Eur J Pediatr. (2022) 181:1727–36. doi: 10.1007/s00431-021-04369-5, 35028728

[29] Duff IT Krolick KN Mahmoud HM Chidambaran V. Current evidence for biological biomarkers and mechanisms underlying acute to chronic pain transition across the pediatric age spectrum. J Clin Med. (2023) 12:5176. doi: 10.3390/jcm12165176, 37629218 PMC10455285

[30] Tan J Shu Y Li Q Liang L Zhang Y Zhang J . Global, regional, and national burden of self-harm among adolescents aged 10-24 years from 1990 to 2021, temporal trends, health inequities and projection to 2041. Front Psych. (2025) 16:1564537. doi: 10.3389/fpsyt.2025.1564537, 40225845 PMC11986636

[31] Al Daajani MM Al-Habib DM Ibrahim MH Al Shewear NA Fagihi YM Alzaher AA . Prevalence of health problems targeted by the national school-based screening program among primary school students in Saudi Arabia, 2019. Healthcare. (2021) 9:1310. doi: 10.3390/healthcare9101310, 34682990 PMC8544408

[32] Dawod MdS Alswerki MN Alelaumi AF AlSamhori JF Rahhal RJ Khraisat L . Evaluation of musculoskeletal complaints, treatment approaches, and patient perceptions in family medicine clinics in a tertiary center in Jordan: a cross-sectional study. BMC Prim Care. (2025) 26:16. doi: 10.1186/s12875-025-02715-2 39838322 PMC11748281

[33] Baniissa W Radwan H Rossiter R Fakhry R Al-Yateem N Al-Shujairi A . Prevalence and determinants of overweight/obesity among school-aged adolescents in the United Arab Emirates: a cross-sectional study of private and public schools. BMJ Open. (2020) 10:e038667. doi: 10.1136/bmjopen-2020-038667, 33310793 PMC7735131

[34] Frosch M Mauritz MD Bielack S Blödt S Dirksen U Dobe M . Etiology, risk factors, and diagnosis of back pain in children and adolescents: evidence-and consensus-based interdisciplinary recommendations. Children. (2022) 9:192. doi: 10.3390/children9020192, 35204913 PMC8870422

[35] Chen H Wu L Zhang Y Liu J Huang R Xie J . Correlation between abnormal posture, screen time, physical activity, and suspected scoliosis: a cross-sectional study. J Orthop Surg Res. (2025) 20:372. doi: 10.1186/s13018-025-05760-w, 40223116 PMC11995642

[36] Feldman DE Nahin RL. National estimates of chronic musculoskeletal pain and its treatment in children, adolescents, and young adults in the United States: data from the 2007-2015 national ambulatory medical care survey. J Pediatr. (2021) 233:212–219. e1. doi: 10.1016/j.jpeds.2021.01.055, 33524388

[37] León-Domínguez A Cansino-Román R Martínez-Salas JM Farrington DM. Clinical examination and imaging resources in children and adolescent back pain. J Child Orthop. (2023) 17:512–26. doi: 10.1177/18632521231215860, 38050588 PMC10693837

[38] Molina-García C López-del-Amo-Lorente A Ramos-Petersen L Martínez-Sebastián C Jiménez-García JD Álvarez-Salvago F . Childhood obesity and its impact on the characteristics of gait stance phases: a cross-sectional study. Eur J Pediatr. (2024) 183:123–34. doi: 10.1007/s00431-023-05268-7, 37843611

[39] Molina-García C Jiménez-García JD Velázquez-Díaz D Ramos-Petersen L López-del-Amo-Lorente A Martínez-Sebastián C . Overweight and obesity: its impact on foot type, flexibility, foot strength, plantar pressure and stability in children from 5 to 10 years of age: descriptive observational study. Children. (2023) 10:696. doi: 10.3390/children10040696, 37189945 PMC10136885

[40] Alahmoradiqashqai A., Full-body biomechanical characterization of children with hypermobile Ehlers-Danlos syndrome during gait and activities of daily living. Odense, Denmark: University of Southern Denmark (2022).

[41] Lamari N Beighton P. "Biomechanical aspects of joint hypermobility". In: Hypermobility in medical practice. Cham, Switzerland: Springer (2023). 47–62.

[42] Billmayer J Jobér A. What’s in it?–schoolbags in the lives of pupils and classrooms. Scand J Educ Res. (2025) 69:884–97.

