# Supplementary figures and images for: Whole-body biomechanical and lifestyle predictors of pediatric musculoskeletal pain: a multi-center cross-sectional study
Source: Front Public Health. 2026 Mar 9;14:1689311. doi: 10.3389/fpubh.2026.1689311 (PMC13006610; doi:10.3389/fpubh.2026.1689311)

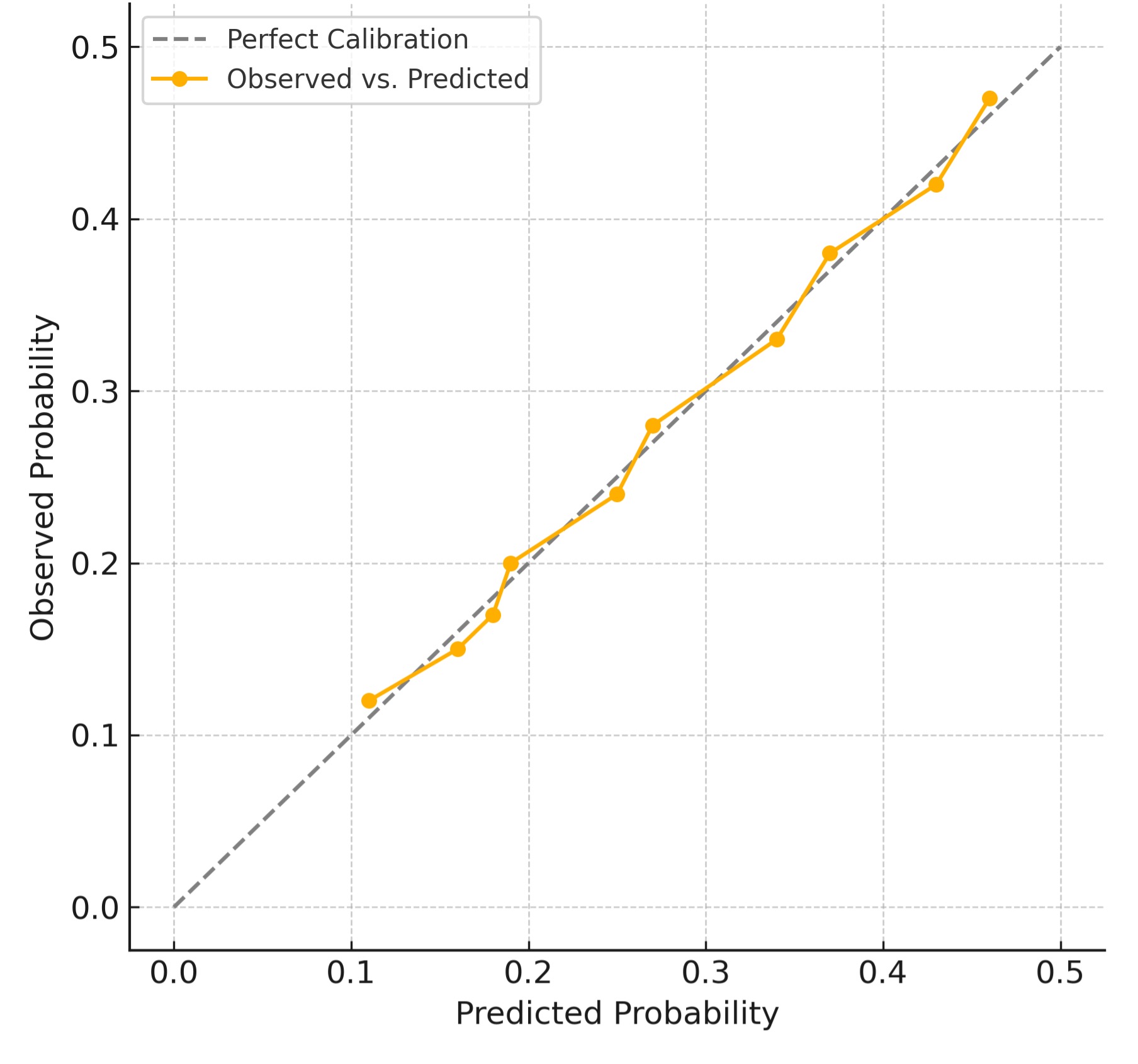

Supplement: Supplementary file 1 [file Image_1.jpeg]
